# Clathrate metal superhydrides under high-pressure conditions: enroute to room-temperature superconductivity

**DOI:** 10.1093/nsr/nwad270

**Published:** 2023-10-31

**Authors:** Ying Sun, Xin Zhong, Hanyu Liu, Yanming Ma

**Affiliations:** Key Laboratory of Material Simulation Methods & Software of Ministry of Education, College of Physics, Jilin University, Changchun 130012, China; State Key Laboratory of Superhard Materials, College of Physics, Jilin University, Changchun 130012, China; Key Laboratory of Material Simulation Methods & Software of Ministry of Education, College of Physics, Jilin University, Changchun 130012, China; State Key Laboratory of Superhard Materials, College of Physics, Jilin University, Changchun 130012, China; Key Laboratory of Material Simulation Methods & Software of Ministry of Education, College of Physics, Jilin University, Changchun 130012, China; State Key Laboratory of Superhard Materials, College of Physics, Jilin University, Changchun 130012, China; International Center of Future Science, Jilin University, Changchun 130012, China; Key Laboratory of Material Simulation Methods & Software of Ministry of Education, College of Physics, Jilin University, Changchun 130012, China; State Key Laboratory of Superhard Materials, College of Physics, Jilin University, Changchun 130012, China; International Center of Future Science, Jilin University, Changchun 130012, China

**Keywords:** clathrate superhydrides, high-temperature superconductivity, high-pressure conditions, crystal structure prediction

## Abstract

Room-temperature superconductivity has been a long-held dream of mankind and a focus of considerable interest in the research field of superconductivity. Significant progress has recently been achieved in hydrogen-based superconductors found in superhydrides (hydrides with unexpectedly high hydrogen contents) that are stabilized under high-pressure conditions and are not capturable at ambient conditions. Of particular interest is the discovery of a class of best-ever-known superconductors in clathrate metal superhydrides that hold the record for high superconductivity (e.g. *T*_c_ = 250–260 K for LaH_10_) among known superconductors and have great promise to be those that realize the long-sought room-temperature superconductivity. In these peculiar clathrate superhydrides, hydrogen forms unusual ‘clathrate’ cages containing encaged metal atoms, of which such a kind was first reported in a calcium hexa-superhydride (CaH_6_) showing a measured high *T*_c_ of 215 K under a pressure of 170 GPa. In this review, we aim to offer an overview of the current status of research progress on the clathrate metal superhydride superconductors, discuss the superconducting mechanism and highlight the key features (e.g. structure motifs, bonding features, electronic structure, etc.) that govern the high-temperature superconductivity. Future research direction along this line to find room-temperature superconductors will be discussed.

## Teaser

This review provides an overview of the current research on clathrate metal superhydrides superconductors, discusses the superconducting mechanism, key features governing high superconductivity, and future directions to find room temperature superconductors.

## INTRODUCTION

**Figure 1.**
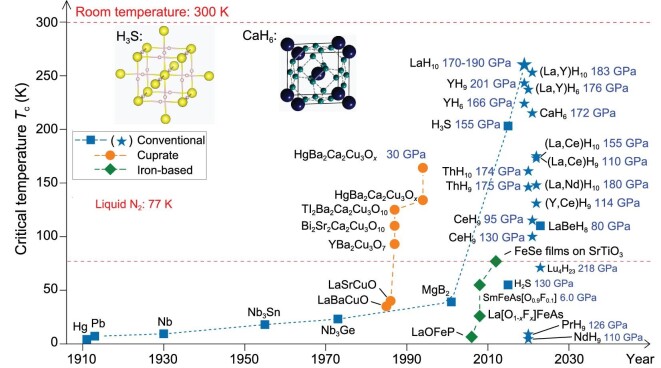
Chronological evolution of the superconducting critical temperature (*T*_c_) for various superconductors. The square, circle and rhombus color blocks represent conventional, cuprate and iron-based superconductors, respectively. In particular, blue stars represent the conventional clathrate metal superhydride superconductors. The pressures required to synthesize these superconductors are represented by blue labels. Inset: crystal structures of covalently bonded hydrogen sulfide superconductor H_3_S [22] (left panel) and clathrate superhydride CaH_6_ [24] (right panel). The yellow, pink, black and green spheres represent the S, H, Ca and H atoms, respectively.

A superconductor exhibits two characteristic physical properties when cooled below its superconducting critical temperature (*T*_c_) where electrical resistance vanishes [1] and magnetic flux fields are expelled from the bulk [2]. Since the first discovery of superconductivity below 4.2 K in solid mercury in 1911, tremendous efforts have been paid to the goal of achieving superconductors that work at ever higher temperatures for practical applications (see Fig. 1 below). Room-temperature superconductors are yet to be achieved and remain a century-long-held dream of mankind.

Intensive superconductivity research has been devoted to the investigation of two families of so-called ‘unconventional’ cuprate and iron-based superconductors, whose superconducting mechanism on the electron pairing is not believed to be mediated by the exchange of phonons [3–6]. In these activities, the highest *T*_c_ of 133 K at ambient pressure [3] was attained in a Hg–Ba–Ca–Cu–O cuprate material whose *T*_c_ was further promoted to 164 K at a high-pressure condition of 31 GPa [4,5], setting the record for *T*_c_ at the time.

On the way to room temperature superconductivity, metallic hydrogen was proposed in 1968 [7] as a potential high-temperature superconductor according to an equation within the superconductive picture of Bardeen-Cooper-Schrieffer (BCS) theory [8] showing that


(1) \begin{eqnarray*}
T_c=1.14\Theta _{D}\exp\! \bigg [-\frac{1}{N(0)V}\bigg ],
\end{eqnarray*}

where Θ_*D*_ is the Debye temperature, *N*(0) is the electron density of states at the Fermi energy and *V* is an effective pairing potential dominated by the attractive electron-phonon coupling interaction. Hydrogen, the most abundant element in the universe having the lightest atomic mass, naturally provides the highest possible Θ_*D*_ and *V* parameters for the solids that are necessary for a high-temperature phonon-mediated superconductivity.

At ambient pressure, solid hydrogen is a wide-gap insulator. It has been suggested that metallization of solid hydrogen would require a strong compression above a pressure of 500 GPa [9–12]. This raises a highly experimental challenge, especially when one deals with hydrogen, the number one element showing the most mobile behavior due to the smallest atomic core among the periodic table of elements. As a result, hydrogen atoms often go into the inside of diamond for a breakdown of the diamond anvil cell, a device for generation of high pressure. As a result, metallic hydrogen has not yet been obtained through a direct compression of solid hydrogen in experiments despite great efforts in the high-pressure research field [13,14].

As an alternative route, hydrogen-containing materials or hydrides play an important role in the pursuit of metallic hydrogen, and its high-temperature superconductivity. The idea was first proposed as early as 1971 [15] in a hypothetic system of Li–F–H and later in 2004 [16] in IV group hydrides (e.g. methane, silane and germane). It is believed that the introduction of non-hydrogen elements into the lattice inevitably causes a chemical pressure to be placed on hydrogen. The resultant metallization pressure of hydrides is significantly reduced compared to that needed for pure hydrogen. High-pressure experimental investigation of metallic hydrides in a lab becomes feasible at the current level of experimental technique.

In recent years, remarkable progress has been achieved in the discovery of high-temperature superconductors in superhydrides stabilized under high-pressure conditions with the established superconductivity *T*_c_ value approaching 260 K (−13 ℃) for LaH_10_ [17–20], a record high *T*_c_ among known superconductors. Major findings are organized into two catalogues: (i) covalently bonded hydrogen sulfide superconductors (e.g. H_3_S with *T*_c_ at ∼200 K) [21–23] and (ii) clathrate metal superhydride superconductors (e.g. CaH_6_ [24–26], YH_6_ [17,27–30], YH_9_ [17,28,30,31] and LaH_10_ [17–20] with *T*_c_ = 215, 220, 240 and 260 K, respectively), as represented by the blue stars in Fig. 1. Particular interest has been placed on the latter class of superconductors found in clathrate metal superhydrides that hold the record high superconductivity and have great potential to be those that superconduct at room temperature. In this peculiar class of clathrate superhydride superconductors, the first such example was theoretically proposed in a calcium superhydride CaH_6_ back to 2012 [24], which was successfully synthesized in 2022 in a lab with a measured high *T*_c_ of 215 K under a pressure of 170 GPa [25,26].

In this article, we review recent progress on the findings of hydrogen-based high-temperature superconductors among superhydrides stabilized under high-pressure conditions, with a particular focus on the family of best-ever-known superconductors found in clathrate metal superhydrides. In the next two sections, we provide a brief overview of the discovery of covalently bonded hydrogen sulfide superconductors, and discuss the CALYPSO crystal structure prediction method and the role it plays in aiding the experimental discovery. Then in the following two sections we respectively discuss metal hydride superconductors at ambient pressure and at high pressures. The latter mainly focuses on clathrate superhydrides, and includes a discussion of the superconducting mechanism and the key features (e.g. structure motifs, bonding features, electronic structure, etc.) that govern the high superconductivity. In the final section, we discuss the future challenge and opportunity for room-temperature superconductors among clathrate metal superhydrides.

## HYDROGEN SULFIDE SUPERCONDUCTORS AT A GLANCE

H_2_S exists as a gas molecule in nature and smells of rotten eggs; it is the only stable stoichiometry compound found at ambient pressure in the hydrogen sulfide system. The work of Li *et al.* [21] is the original literature proposing sulfur hydride as a superconductor under high-pressure conditions.

Since 2014 when Li *et al.* made the first attempt to predict high-temperature superconductivity in sulfur dihydride [21], there has been much less interest in this system since it was believed to dissociate into its constituent elements of sulfur and hydrogen under pressure [32,33]. Li *et al.*’s extensive structure-searching simulation on hydrogen sulfide via the CALYPSO method [34,35] found that earlier proposed elemental dissociation would not occur, and a structure with a high superconductive potential consisting of 2(SH_3_) units was predicted for H_2_S with a theoretical *T*_c_ reaching 80 K at a pressure of 160 GPa [21].

This theoretical work [21] initiated the practical work by Eremets’ group [23] where H_2_S compressed in a diamond anvil cell was found to exhibit two astonishing superconductive states: (i) the one prepared at low temperature has a *T*_c_ of 30–150 K, in high accordance with the predicted H_2_S superconductor [21]; (ii) the one annealed at room temperature exhibits an unexpected high *T*_c_ at 180–203 K, surpassing the earlier *T*_c_ record of 164 K [5] set by cuprate. The latter one has been ascribed to be H_3_S through a stoichiometric change via a decomposition of H_2_S into H_3_S+S [36], where H_3_S is a known stoichiometry of (H_2_S)_2_H_2_ that has already been synthesized at a pressure of 7 GPa [37] and was later theoretically predicted to be a 200-K superconductor with a cubic structure (space group $Im\bar{3}m$) at megabar pressures [22]. In this cubic structure, each pair of S atoms symmetrically accommodates an atomic H between them, forming robust six-fold polar covalent S–H bonds (as shown in the inset of Fig. 1). The observed superconductivity in H_3_S shows a strong isotopic effect pointing toward a phonon-mediated pairing mechanism [23].

The findings of hydrogen sulfide superconductors at a megabar pressure condition mark a milestone in superconductivity history and are a result of joint theoretical and experimental efforts, where theory plays a critical role in guiding the experimental exploration.

## CALYPSO CRYSTAL STRUCTURE PREDICTION METHOD AND ITS ROLE IN AIDING EXPERIMENTAL DISCOVERY

Research on unknown superhydrides presents a challenge, as they can only be produced under megabar pressure conditions where limited information is available regarding their compositions and crystal structures. Moreover, owing to the weak X-ray scattering of hydrogen, the exact position of hydrogen is hardly determined by X-ray diffraction experiments. All these difficulties call for advanced theory that can predict the crystal structures of superhydrides in a reliable manner with only the chemical composition given. Recently developed theoretical crystal structure prediction methods [38,39] have taken center stage for this purpose, due to their trustworthy predictive power regarding compositions and structures. Over the years, techniques such as random search, genetic algorithm and swarm intelligence methods have evolved to become the preferred choices for computational discovery of structures [38,39].

One may recall that crystal structure prediction presents a challenging, NP-hard problem in the minimization of the high-dimensional potential-energy surface (PES). To deal with this challenging problem, the CALYPSO (crystal structural analysis by particle swarm optimization) structure prediction method has been developed by the Ma group [34]. The method adopts a heuristic numerical solution scheme that is an optimal compromise between global exploration and local exploitation of the potential energy surface, making it particularly suited for crystal structure prediction. A number of algorithms has been devised in the method: (i) a symmetry classification searching strategy for high-coverage sampling of the potential energy surface; (ii) a bond characterization matrix for fingerprinting the structures and dividing the entire PES into a set of simpler fragments that are easier to explore and, most importantly, (iii) a swarm intelligence algorithm for rapid location of the energetically most stable structure by swarm-directed smart learning of optimal structures. The CALYPSO method has been coded into the same-name software [35] that is freely available for academic users, and it has proven a highly efficient and accurate tool for crystal structure prediction.

Given chemical compositions, CALYPSO can predict structures, in an intelligent and automatic way, for two- or three-dimensional crystals, nanoclusters and nanoparticles, protein molecules, reconstructed surfaces and interfaces, etc. The proof of the method’s generality and reliability is in its utilization: up to now (June 2023), CALYPSO has been widely used in the world by more than 4000 researchers from 74 countries. Use of CALYPSO has generated many groundbreaking discoveries dedicated to high-pressure science, including the long-puzzled *oC*40 phase structure of semiconducting lithium, chemical compounds of Fe/Ni_3_Xe, the substitutional alloy structure of Bi_2_Te_3_, the atomic structure of solid oxygen, counterintuitive compounds of H_3_O and Ca_3_O, the polymeric N_10_ structure, the superhard cubic phase of BC_3_, etc. [39].

CALYPSO has played an important role in the design of hydrogen-based superconductors under high-pressure conditions. Besides the above-mentioned prediction of metallic and superconducting structures of H_2_S [21], CALYPSO has been used to make breakthrough predictions on a class of clathrate metal superhydride superconductors (e.g. CaH_6_ [24], YH_6_ [27], YH_9_ [17], LaH_10_ [17,18], etc.) that have received subsequent experimental confirmation. With the development of the crystal-structure searching methods, future design of room-temperature superhydride superconductors becomes feasible.

## METAL HYDRIDE SUPERCONDUCTORS AT AMBIENT PRESSURE

The first hydride superconductor ever reported in 1970 was Th_4_H_15_ with a *T*_c_ of 8 K at ambient pressure [40]. Later on, other binary metal hydride superconductors of PdH [41] and NbH_0.69_ [42] were also reported with *T*_c_ values of ∼10 K. Several ternary metal hydride superconductors (e.g. HfV_2_H [43] and Pd_0.55_Cu_0.45_H_0.7_ [44] with *T*_c_ values of 4.8 and 16.6 K, respectively) were also synthesized.

**Figure 2.**
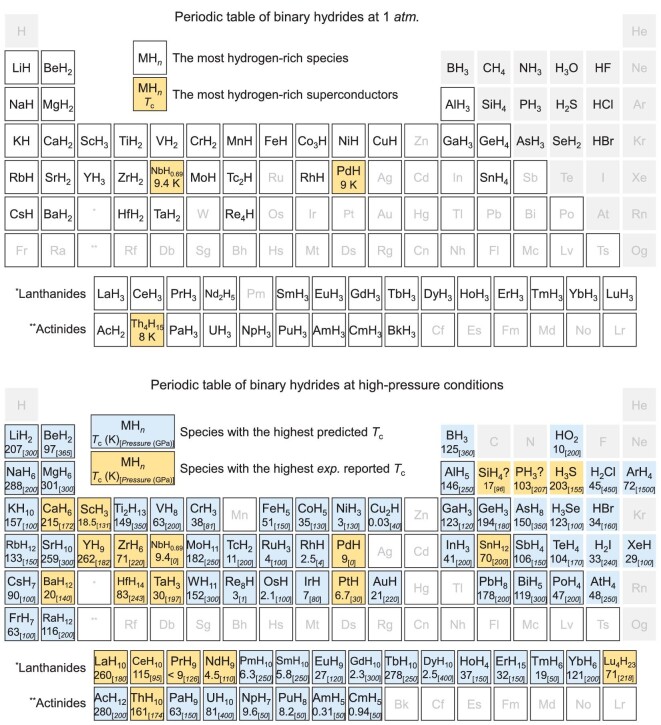
Periodic table of experimental binary hydrides synthesized under ambient pressure (1 *atm.*, top panel) or high-pressure conditions (bottom panel). We use boxes with and without a black border to respectively distinguish between metal elements and covalent elements. Only the most hydrogen-rich species or those with the highest *T*_c_ (measured *T*_c_ values are preferred to the estimated ones) are listed, with theoretical predictions given a blue background and experimental results given an orange background. The related data were collected from Table 1, the Inorganic Crystal Structure Database [46] and other excellent reviews [47–49].

**Figure 3.**
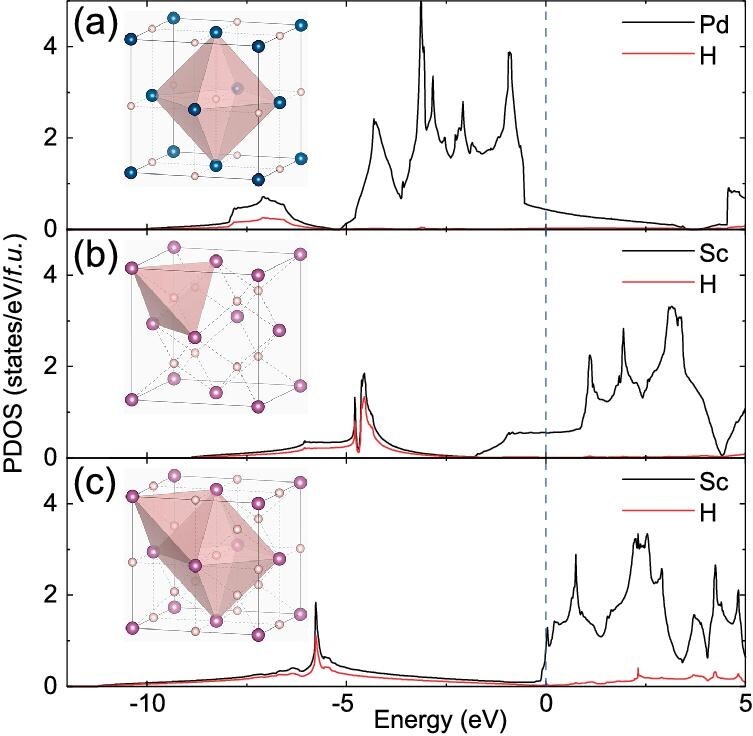
Crystal structures (inset) and partial electron density of states (PDOS) of *face-centered-cubic*-structured (a) PdH, (b) ScH_2_ and (c) ScH_3_ at ambient pressure. The blue, purple and pink spheres represent Pd, Sc and H atoms, respectively. Most H atoms occupy the interstitial octahedral (*O*) or tetrahedral (*T* ) sites of the metal lattice. Solid black and red lines represent PDOS of metal and H, respectively. Blue dotted lines represent Fermi levels.

It is worth noting that all these ambient-pressure metal hydride superconductors possess low superconductivity (<16.6 K). The top panel of Fig. 2 lists the most hydrogen-rich hydrides achieved at ambient pressure. It is seen that H contents in these ambient-pressure metal hydrides are generally low with a metal/H ratio larger than 1/3. In the crystal structures (Fig. 3), H atoms typically occupy the interstitial octahedral (*O*) or tetrahedral (*T*) sites of the closely packed metal lattice. In order to understand the physical origin for the low superconductivity at ambient pressure, the electron density of states of PdH [41], ScH_2_ [45] and ScH_3_ [45] are calculated, as shown in Fig. 3. It is found that hydrogen electrons are localized at a low-lying energy level, and do not contribute to the Fermi level. As a result, hydrogens in these low-H-content hydrides are not expected to play an important role in the superconductivity. It is worth noting that two properties of metal hydrides are crucial to achieving H-dominated high-*T*_c_ superconductivity: a large H-derived density of state at the Fermi level and large modifications of the electronic structure in response to the motions of H atoms (electron-phonon coupling) [17,38]. It is apparent that these ambient-pressure metal hydrides are not good candidates for the H-dominated superconductors.

**Figure 4.**
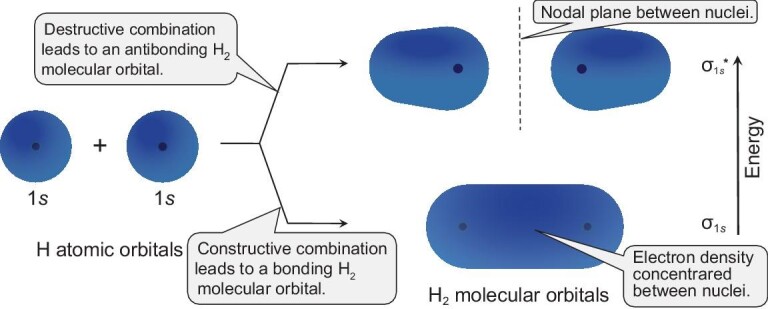
The formation of H_2_ molecular orbitals from two H atomic orbitals based on molecular orbital theory, where the lower- (higher-)energy molecular orbital σ_1*s*_ ($\sigma _{1s}^{*}$) corresponds to the bonding (antibonding) behavior with electron density concentrated between (behind) the H nuclei.

From a chemical point of view, it is not unexpected to see such a negligible hydrogen electron contribution to the Fermi level in these hydrides. Metal hydrides can be regarded as M^+^ cation-doped solid hydrogen (H_2_). Once there is a formation of hydrides, valence electrons of metal atoms will transfer to H_2_ molecules in the solid due to the large difference in electronegativities of hydrogen and metal atoms. The resultant electron occupancy of the antibonding $\sigma _{1s}^*$ orbitals of H_2_ molecules (Fig. 4) will lead to the dissociation of molecules into hydrogen atoms forming ionic Pd^+^…H^−^, Sc$_2^+$…2H^−^ and Sc$_3^+$…3H^−^ bonding states in PdH, ScH_2_ and ScH_3_, respectively. As a result, hydrogen electrons are confined in H^−^ states at deep energy levels far away from the Fermi level (Fig. 3).

Recently, a ternary N-doped lutetium hydride [50] was claimed to exhibit a remarkably high *T*_c_ of 294 K (21 ℃) at a near-ambient pressure of 1 GPa. However, a number of subsequent experiments [51–53] that were able to reproduce the as-synthetic ternary product of N-doped lutetium hydride found no superconductivity at all for such a sample. From the current results achieved, it is suggested that such a claimed high superconductivity in an N-doped lutetium hydride is unlikely to be true.

## CLATHRATE METAL SUPERHYDRIDE SUPERCONDUCTORS AT HIGH PRESSURES

Pressure as a thermodynamical variable can profoundly modify electronic orbitals and bonding patterns of hydrogen and metal atoms. It is thus a powerful tool for the creation of exotic metal superhydrides (Fig. 2) that are not accessible at ambient conditions. As can be seen from Fig. 2, many new binary superhydrides appear with extremely high H content under high-pressure conditions. This opens a research avenue for finding high-temperature superconductors in these newly formed superhydrides.

A large number of superhydrides have been experimentally synthesized, some of which have been confirmed to be high-*T*_c_ superconductors, as listed in Table 1. Among various superhydrides, clathrate metal superhydrides take center stage since they exhibit the highest-*T*_c_ values (up to 250–260 K) known thus far.

**Table 1.** Measured superconducting transition temperature values (*T*_c_ in kelvins) of experimentally confirmed hydrides under ambient pressure and high-pressure conditions (P in gigapascals).

Class	Compound	P (GPa)	*T*_c_ (K)	Ref.
Hydride at 1 *atm.*	Th_4_H_15_	0	8.35	[40]
	PdH	0	9	[41]
	Pd_0.55_Cu_0.45_H_0.7_	0	16.6	[44]
	HfV_2_H	0	4.8	[43]
	NbH_0.69_	0	9.4	[42]
Covalent hydride	SiH_4_?	96	17	[72]
	H_2_S	130	55	[23]
	H_3_S	155	203	[23,73]
	H_3_P?	207	103	[74]
Ionic hydride	BaReH_9_	91	7	[75]
	Li_5_MoH_11_	160	6.5	[76]
	PtH	30	6.7	[77]
	ScH_3_	131	18.5	[78]
	ZrH_3_	9	11.6	[79,80]
	LuH_3_	122	12.4	[78]
	TaH_3_	197	30	[81]
	Zr_4_H_15_	40	4	[79]
	Hf_4_H_15_	23	4.5	[80]
Quasi-H_2_-contained hydride	YH_4_	155	88	[30,82]
	SnH_4_	180	72	[83]
	(La,Y)H_4_	111	92	[84]
	ZrH_6_	220	71	[85]
	YH_7_	162	29	[30]
	CeH_9_	88	57	[86]
	SnH_12_	200	70	[87]
	BaH_12_	140	20	[88]
	HfH14	243	83	[89]
CaH_6_-type clathrate	CaH_6_	172	215	[25,26]
	YH_6_	166	224	[28–30]
	EuH_6_	152	–	[66]
	(La,Y)H_6_	176	237	[71]
YH_9_-type clathrate	YH_9_	201	243	[28,30,31]
	CeH_9_	130	100	[86]
	PrH_9_	126	<9	[90]
	NdH_9_	110	4.5	[91]
	EuH_9_	170	–	[66]
	ThH_9_	170	146	[92]
	(Y,Ce)H_9_	114	131	[93]
	(La,Ce)H_9_	100	176	[94]
LaH_10_-type clathrate	LaH_10_	188	260	[19,20]
	CeH_10_	95	115	[86]
	ThH_10_	174	161	[92]
	(La,Y)H_10_	183	253	[71]
	(La,Ce)H_10_	155	175	[95]
	(La,Nd)H_10_	180	148	[96]
Ba_8_Si_46_-type clathrate	Ba_8_H_46_	27	–	[97]
	Eu_8_H_46_	86	–	[98]
	La_4_H_23_	96	–	[99]
	Lu_4_H_23_?	218	71	[100]
LaBH_8_-type hydride	LaBeH_8_	80	110	[101]

### CaH_6_: the first clathrate superhydride

**Figure 5.**
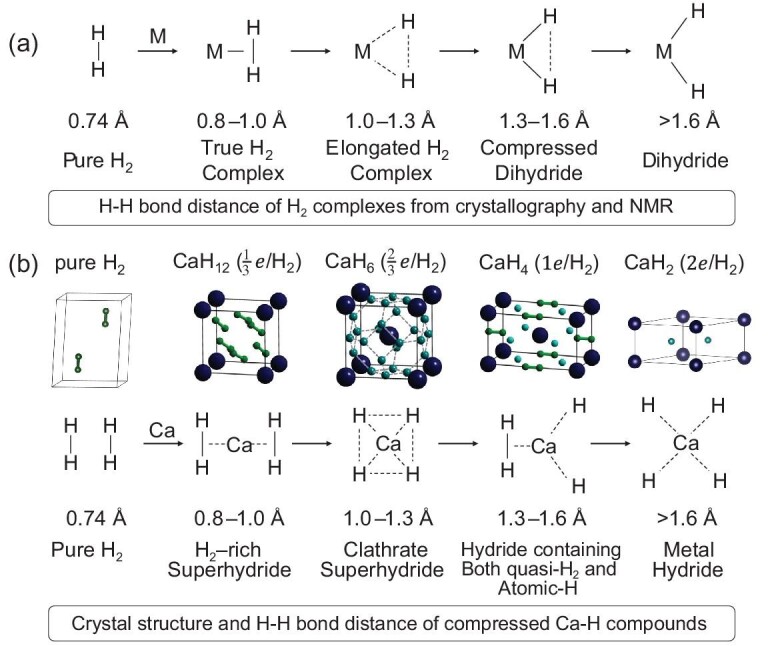
(a) The entire reaction coordinate for the activation of H_2_ on a metal as a function of the degree of backdonation within the large regime of hundreds of LnM-H_2_ complexes, Adapted with permission from [54]. Copyright 2007 American Chemical Society. (b) Several typical H-motifs of compressed metal. The corresponding crystal structures are illustrated with a Ca–H system as an example. H–H bond distance (*d*_H-H_) of H_2_ complexes (from crystallography and NMR) or H-motifs varying from 0.74 to >1.6 Å are shown.

At ambient pressure, the only thermodynamically stable compound in the Ca–H system has a stoichiometry of CaH_2_ that adopts a cotunnite-type structure and is not a superconductor (we refer the reader to our discussion on ambient-pressure hydrides). In 2012, through a structure search study on a mixture of Ca + H_2_ using the CALYPSO method [34,35], we theoretically explored other calcium hydrides with higher hydrogen contents that can be stabilized under high-pressure conditions [24]. It was predicted that, besides CaH_2_, three new stoichiometric calcium hydrides appear (Fig. 5(b)), CaH_4_, CaH_6_ and CaH_12_, that are thermodynamically stable at the megabar pressure regime.

CaH_4_ is not a superconductor and has a tetragonal *I*4/*mmm* structure (Fig. 5(b)) where Ca adopts a body-centered arrangement and H takes a mixed chemical form of monoatomic H and molecular H_2_ [24]. Following our prediction, this proposed CaH_4_ has been subsequently synthesized by two independent experimental works [55,56]. CaH_12_ has a rhombohedral structure consisting entirely of molecular H_2_ units (Fig. 5(b)) that is not a good candidate for high-*T*_c_ superconductors [24].

**Figure 6.**
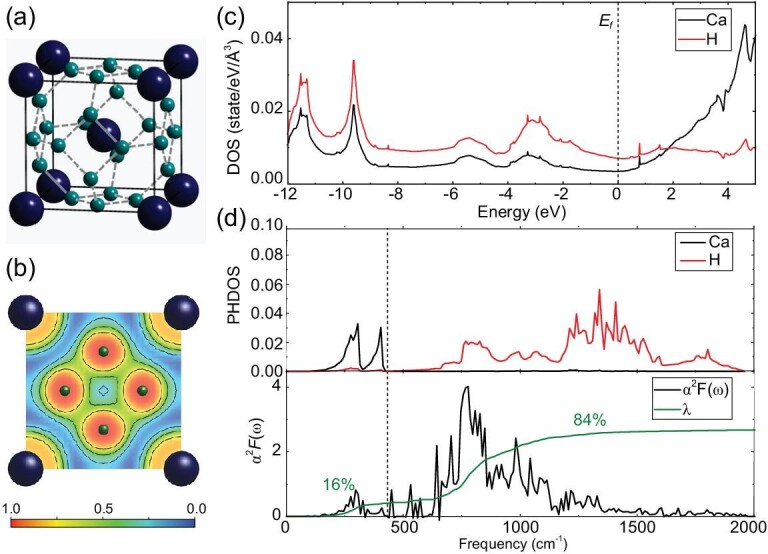
(a) Crystal structure, (b) the electron localization function, (c) the partial electron density of states and (d) projected phonon densities of states as well as the isotropic Eliashberg spectral function α^2^*F* (ω) and EPC parameter λ(ω) of CaH_6_ at 150 GPa [24]. The small and large spheres in (a, b) represent H and Ca atoms, respectively.

Of particular interest is the prediction of a peculiar clathrate-structured CaH_6_ that adopts a body-centered cubic structure (space group $Im\bar{3}m$; Figs 5(b) and 6(a)) where 24 H atoms form a perfect cage (eight hexagons plus six squares) with Ca at the center of the cage [24]. Within the H cage, there is clear charge localization (Fig. 6(b)) between nearest-neighboring H atoms and analysis of the electron localization function indicates that there is a weak covalent interaction between H atoms. It is interesting to note that all the nearest H–H distances are equal to 1.24 Å at 150 GPa. From the calculated partial electron density of states (Fig. 6(c)), we see that H electrons dominate the density of states at the Fermi level in this structure. As we have discussed in the above context, the dominant contribution of H electrons to the Fermi level is the most significant signature for the H-based superconductor. Here, CaH_6_ must be the one that we really want in the hunt of a H-based high-*T*_c_ superconductor. Indeed, a realistic electron-phonon coupling calculation on CaH_6_ gave a predicted exceptionally high *T*_c_ of 220 K at 150 GPa [24]. As expected, the H cage is found to be crucial to the superconductivity since it contributes most (84%) to the electron-phonon coupling parameter (Fig. 6(d)).

Although CaH_6_ is the first-ever clathrate-type superhydride superconductor proposed in the field, its experimental synthesis has been regarded as one of the most challenging tasks. Previous attempts [55,56] on the synthesis use a mixture of Ca + H_2_ or CaH_2_ + H_2_ as precursors. Unfortunately, this synthetic route leads to the easy formation of low-H content CaH_4_ at a low-pressure region and further formation of high-H CaH_6_ at the higher-pressure regime is kinetically hindered. Thanks to the concept of using ammonia borane [57–59] as the hydrogen source where it decomposes into boron mononitride and hydrogen at high-temperature conditions, one can control the hydride synthesis at a desirable pressure condition. Very recently, after 10 years of continuous effort, two independent experimental works [25,26] reported the successful syntheses of as-predicted clathrate-structured CaH_6_ by using a mixture of Ca and ammonia borane as precursors. Experimentally measured *T*_c_ values for CaH_6_ are 215 K at 172 GPa [25] and 210 K at 160 GPa [26], respectively.

Since we have various calcium hydrides at hand, it is possible to derive a general picture of the evolution of the chemical bonding of hydrogen to understand the formation mechanism of various calcium hydrides under pressure, as depicted in Fig. 5(b). Ca was introduced into solid molecular hydrogen as a dopant to achieve metallic hydrogen. As a result, there will be inevitable charge transfer from Ca into H_2_ molecules. The accepted electrons by each H_2_ molecule will occupy the antibonding $\sigma _{1s}^{*}$ orbital (Fig. 4) since each H_2_ molecule already has a filled σ_1*s*_ bond. The occupancy of this $\sigma _{1s}^{*}$ orbital will lead to a weakening of the H–H bond, which in turn lengthens the H–H bond length and ultimately results in the complete dissociation of the H_2_ molecule. The existence of monatomic H and H_2_ units in the structures is contingent on the number of electrons each H_2_ molecule accepts. If we assume that the two valence electrons of each Ca atom are fully ‘ionized’ and taken up by H_2_ molecules, then the accepted electrons per H_2_ for CaH_12_, CaH_6_, CaH_4_ and CaH_2_ are $\frac{1}{3} e$, $\frac{2}{3} e$, 1*e* and 2*e*, respectively.

As for CaH_12_, each H_2_ molecule accepts $\frac{1}{3} e$ without severing the bond, but rather results in a bond elongation from 0.74 to 0.8–1.0 Å. In CaH_6_, acceptance of $\frac{2}{3} e$ per H_2_ molecule leads to the formation of the clathrate structure [24], where the H–H distance is in the range 1.0–1.3 Å and H–H is weakly covalently bonded. In CaH_4_, there are two H_2_ formulas in each unit cell and each H_2_ molecule accepts 1*e*. Given that two H_2_ molecules are preserved, the remaining two H_2_ molecules accommodate four ‘excess’ electrons into their $\sigma _{1s}^{*}$ orbital. This process disrupts the molecules into monatomic hydrogen, with the H–H distances in the range 1.3–1.6 Å. In CaH_2_, each H_2_ molecule accepts 2*e*. As a result, H_2_ molecules dissociate fully into monatomic H.

We point out that H–H bonding features in calcium hydrides discovered under high-pressure conditions show excellent agreement with those in metal-inclusion H_2_ complexes (LnM-H_2_) at ambient pressure [54] (Fig. 5(a)) that have been well established by nuclear magnetic resonance experiments, as depicted in Fig. 5(a). This analogue for H–H bonding behaviors between calcium superhydrides stabilized under high-pressure conditions and H_2_ complexes at ambient pressure is not accidental, but it rather reflects the true physics of hydrogen. We further emphasize that the current analysis of hydrogen bonding is also applied to the understanding of chemical bonding in other superhydrides and it is thus not necessary to discuss again in the context below to avoid any repetition.

### Other CaH_6_-type clathrate superhydrides

As a follow up to the theoretical proposal of clathrate CaH_6_ as a high-temperature superconductor [24], other CaH_6_-type clathrate superhydrides have also been predicted under high-pressure conditions, including, e.g. MgH_6_[60], YH_6_[17,27], ScH_6_[17,61,62], PuH_6_[63], TbH_6_[64], EuH_6_[65,66] and (Yb/Lu)H_6_[67,68]. Among these, YH_6_[17,27] has been experimentally synthesized under high-pressure conditions[28–30] with measured *T*_c_ values of up to 224 K at 166 GPa. These experimental works directly confirmed the theoretical prediction [17,27] of the clathrate structure and the high superconductivity above 200 K of YH_6_. A strongly correlated system of clathrate EuH_6_ has also been synthesized [66]; however, its superconductivity is absent due to the existence of magnetic properties in the system.

Several CaH_6_-type alloyed clathrate superhydrides in ternary systems have been proposed by using the idea of partial substitution of Ca in CaH_6_ or Y in YH_6_ with alternative metal elements, e.g. CaYH_12_ [69] and YMgH_12_ superhydrides [70]. It is worth noting that a ternary clathrate alloyed (La,Y)H_6_ superconductor has been experimentally synthesized with a measured *T*_c_ of 237 K at a pressure of 176 GPa [71].

### YH_9_-type clathrate superhydrides

**Figure 7.**
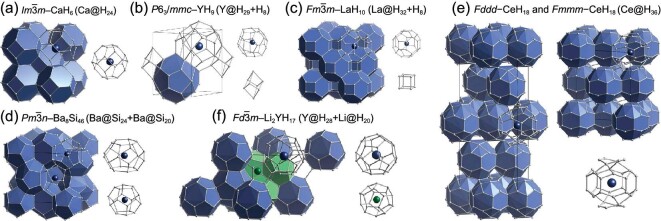
Crystal structures of several typical clathrate superhydrides and their H-motifs. (a) $Im\bar{3}m$–CaH_6_, which is composed of Ca-centered H_24_ cages. (b) *P*6_3_/*mmc*–YH_9_, which is composed of Y-centered H_29_ cages and distorted H_8_ cubes. (c) $\textit{Fm}\bar{3}m$–LaH_10_, which is composed of La-centered H_32_ cages and H_8_ cubes. (d) Ba_8_Si_46_-type $Pm\bar{3}n$–Ba_8_H_46_, which is composed of Ba-centered H_24_ and Ba-centered H_20_ cages. (e) *Fddd*– and *Fmmm*–CeH_18_, which are composed of Ce-centered H_36_ cages. (f) $Fd\bar{3}m$–Li_2_YH_17_, which is composed of Y-centered H_28_ and Li-centered H_20_ cages. The small and large spheres represent H and metal atoms, respectively.

In 2017, Peng *et al.* [17] theoretically proposed that clathrate structures are commonly formed in rare-earth superhydrides with stoichiometries besides CaH_6_, with even higher hydrogen contents (e.g. YH_9_ and LaH_10_). The structure of YH_9_ [17] in space group *P*6_3_/*mmc* can be regarded as a variant of CaH_6_, although the number of hydrogen atoms in the hydrogen cage has been enlarged from 24 atoms in CaH_6_ to 29 atoms in YH_9_. In this H_29_ cage (Fig. 7(b)) where one Y atom sits at the center of the cage, there are six quadrilaterals, six pentagons and six regular hexagons [17]. YH_9_ was computed to exhibit superconductivity with *T*_c_ reaching room temperature at 269 K at a pressure of 150 GPa. Soon after this theoretical prediction, two independent experiments successfully synthesized the clathrate-structured YH_9_ and reported measured *T*_c_ values of 262 K at 182 GPa[31] and 243 K at 201 GPa[28].

Subsequent experimental efforts further successfully synthesized other YH_9_-type rare-earth clathrate superhydrides in CeH_9_[86], PrH_9_[90], NdH_9_[91] and EuH_9_[66] with the measured highest *T*_c_ reaching 100 K for CeH_9_[86]. These experimental results gave direct confirmation of theoretical predictions by Peng *et al.* [17]. A YH_9_-type clathrate superhydride has also been reported in ThH_9_ among actinide hydrides with the measured *T*_c_ reaching 146 K at a pressure of 170 GPa[92].

Recently, two YH_9_-type ternary alloyed superhydrides of (La,Ce)H_9_[94] and (Y,Ce)H_9_[93] have been synthesized via random substitution of half Ce by La and Y, respectively. These two works demonstrated that substitutional alloying could act as an effective tool for substantially enhancing superconductivity since giant *T*_c_ enhancements are evidential. Superconductivity of 100 K in parent CeH_9_ has been enhanced to 178 and 131 K in resultant child (La,Ce)H_9_ and (Y,Ce)H_9_, respectively, after substitutional alloying.

It is worth noting that the notably low synthesis pressures of 100–130 GPa for CeH_9_, (La,Ce)H_9_ and (Y,Ce)H_9_ are believed to result from the particularly strong chemical pressure exerted by the delocalized 4*f* electrons of Ce. This suggests that other rare-earth hydrides with a similar delocalized *f* character may also be stable at relatively low pressures[102].

### LaH_10_-type clathrate superhydrides

In the same work for the proposal of YH_9_-type superhydrides, Peng *et al.* [17] also theoretically proposed a class of clathrate rare-earth superhydrides in a stoichiometry of LaH_10_ with predicted *T*_c_ values as high as 288 K for LaH_10_ at 200 GPa and 303 K for YH_10_ at 400 GPa. The structure of LaH_10_ in space group $Fm\bar{3}m$ can also be regarded as a variant of the CaH_6_-type structure, where the number of hydrogen atoms in the hydrogen cage has been enlarged from 24 atoms in CaH_6_ to 32 atoms in LaH_10_. The H_32_ cage (Fig. 7(c)) is composed of six squares and 12 regular hexagons [17]. Meanwhile, Liu *et al.* also independently predicted the clathrate-structured LaH_10_ and YH_10_ by a systematic exploration of the structures and superconductivities of La–H and Y–H systems under high-pressure conditions [18]. The as-predicted LaH_10_ has been successfully synthesized in two independent experiments [19,20] with measured *T*_c_ values of 250–260 K at pressures of 170–185 GPa.

The successful synthesis of LaH_10_ at pressures <200 GPa corroborated recent theoretical calculations by taking quantum effects into account [103] where the classical *ab initio* calculations [17,18] predicted structural distortions in the LaH_10_ below ∼230 GPa. The inclusion of zero-point energy has a clear effect on the stabilization of the $Fm\bar{3}m$ phase at a pressure regime as low as 129 GPa [103].

It is worth mentioning that the synthesis of LaH_10_ created a *T*_c_ record of 250–260 K (Fig. 1) approaching room temperature and ignited the hope of finding of room-temperature superconductivity in clathrate superhydrides. Subsequently, LaH_10_-type clathrate superhydrides including CeH_10_ [86] and ThH_10_[92] have also been experimentally synthesized with measured *T*_c_ values of 115 and 161 K, respectively. Substitutionally alloyed ternary LaH_10_-type clathrate superhydrides in (La,Y)H_10_[71], (La,Ce)H_10_[95] and (La,Nd)H_10_[96] have also been experimentally synthesized with measured *T*_c_ values of 253, 175 and 148 K, respectively.

### Ba_8_Si_46_-type clathrate superhydrides

It is known that clathrate structures often appear in a variety of silicon/germanium-based materials. Ba_8_Si_46_ is one such good example, in the structure (Fig. 7(d)), and it is the first clathrate superconductor found in a bulk phase [104] with a measured *T*_c_ of 8 K at ambient pressure.

Several Ba_8_Si_46_-type clathrate superhydrides including Ba_8_H_46_ [97], Eu_8_H_46_ [98], La_4_H_23_[99] and Lu_4_H_23_[100] have been experimentally synthesized, among which Lu_4_H_23_ has an observed *T*_c_ value up to 71 K at 218 GPa. No superconducting properties were experimentally studied for La_4_H_23_ and Ba_8_H_46_, although they are expected to be high-*T*_c_ superconductors. Eu_8_H_46_ is not a superconductor since it was predicted to exhibit a ferromagnetic property caused by the local unpaired *f* electrons of the Eu atom.

The theoretical proposal for thermodynamically stable Ca_8_H_46_ and Sr_8_H_46_ [105] is interesting; their predicted *T*_c_ at 200 GPa are 214 and 203 K, respectively. In addition, by performing a combination of high throughput screening and structural search, An *et al.* [106] predicted a thermodynamically stable Ba_8_Si_46_-type superhydride of LiNa_3_H_23_ that exhibits an extraordinarily high *T*_c_ of 310 K at 350 GPa.

### CeH_18_-type clathrate superhydrides

Very recently, a new class of extremely hydrogen-rich CeH_18_-type clathrate superhydrides with a stoichiometry of CeH_18_ were theoretically proposed in rare-earth/actinide superhydrides [107]. These peculiar superhydrides are composed of H_36_ cages (Fig. 7(e)), the largest cage for a known clathrate superhydride structure. This CeH_18_-type clathrate superhydride forms a crystal structure with either the *Fddd* or *Fmmm* space group. In this structure, H_36_ cages are interconnected by a 6H_6_ ribbon-ring structure. Two undulating H_6_ hexagons are positioned above and below this structure, with bridge bonds linking the H_6_ hexagons to the 6H_6_ ribbon ring (as shown in Fig. 7(e)). First-principles calculations [107] for different CeH_18_-type clathrate superhydrides predict diverse *T*_c_ values among the same stoichiometry. Among these extreme superhydrides, CeH_18_ and ThH_18_ are particularly noteworthy. They display superconductivity above room temperature, with *T*_c_ values peaking at 330 K at 350 GPa and 321 K at 600 GPa, respectively. These represent the highest predicted *T*_c_ values among all known thermodynamically stable superhydrides. Future experiments are highly desirable for the synthesis of this class of CeH_18_-type clathrate superhydrides to search for room-temperature superconductors.

### Ternary clathrate superhydrides

Binary superhydrides have been exhaustively investigated both theoretically and experimentally [47]. The quest for high-*T*_c_ superconductors among superhydrides has recently evolved, moving beyond the realm of binary compounds and shifting focus towards ternary ones, which offer a vast array of material types and configurations. Ternary superhydrides can enjoy advantageous properties through tuning of two non-hydrogen elements in structures, leading to superior superconducting properties beyond those of binary hydrides. Earlier theoretical works [108,109] have revealed promising ternary superhydrides that superconduct at or above room temperature. However, the challenge remains to attain stoichiometric ternary compounds with a well-resolved crystal structure that can host high-temperature superconductivity above 100 K. Although several examples of ternary substitutionally alloyed clathrate superhydrides (e.g. (La,Ce)H_9_ and (Y,Ce)H_9_) have been discussed in the aforementioned text where the primary focus is placed on enhancing superconductivity through elemental substitution on known binary clathrate hydride superconductors, these activities have not offered any new prototype ternary structure for high-temperature superconductivity.

On the way to finding room-temperature superconductors among ternary superhydrides, a significant research question in this field is how to stabilize a superhydride that incorporates as many hydrogen atoms as possible, without forming any H_2_ molecules within the lattice, in order to achieve a hydrogen-dominated electron density of states at the Fermi surface [17,38]. A useful strategy is to introduce extra electrons via metal doping into H_2_-rich binary superhydrides [110], as has been demonstrated in the design of clathrate-structured ternary Li_2_MgH_16_[110] and Li_2_Y/LaH_17_[111]. Li_2_MgH_16_ has been predicted to exhibit ‘hot’ superconductivity with a *T*_c_ value of up to 473 K at 250 GPa, which is the highest predicted *T*_c_ among all known superhydride superconductors [110]. The Li_2_Y/LaH_17_ clathrate structure comprises Li-centered H_20_ cages and Y/La-centered H_28_ cages, where each H_20_ or H_28_ cage consists of 12 pentagons or 12 pentagons and four hexagons (Fig. 7(f)). It is worth noting that the estimated *T*_c_ values of Li_2_YH_17_ and Li_2_LaH_17_ are relatively low with maximum values of 108 K at 200 GPa and 156 K at 160 GPa, respectively [111].

The challenge of achieving room-temperature superconductivity at significantly lower pressures is a recognized issue. A strategy to address this challenge is to use small-radius elements (e.g. B or Be) and hydrogen to achieve ternary superhydride superconductors with alloy backbones [112]. By employing this approach, a range of LaBH_8_-type ternary superhydrides with a ‘fluorite-type’ backbone have been proposed [112–114], among which LaBeH_8_ [112] is anticipated to be a thermodynamically stable phase above 98 GPa and dynamically stable down to 20 GPa with a high *T*_c_ of 185 K. Very recently, LaBeH_8_ has been successfully synthesized with a measured *T*_c_ of up to 110 K at 80 GPa [101]. LaBeH_8_ is the first experimental realization of an archetype ternary prototype with exact stoichiometry, well-resolved structure and *T*_c_ beyond 100 K.

### Clathrate frameworks at ambient pressure

Hydrogen clathrate structures in superhydrides stabilized under high-pressure conditions are a class of peculiar structures quipped for hydrogen-based superconductors. As we have discussed above, the clathrate structure is the key to create the observed high superconductivity in superhydrides that holds the record high-*T*_c_ values among superconductors known thus far. However, these hydrogen clathrate structures can only be stabilized at megabar pressures and are not capturable at ambient pressure upon the release of pressure. For any practical application, it is essential to discover superconductors at ambient pressure.

From a structure point of view, we find two kinds of analogous materials at ambient pressure: (i) CaH_6_-, Ba_8_H_46_- and Li_2_YH_17_-type superhydrides are structurally equivalent to the type-VII, type-I and type-II silicon-based clathrate structures, respectively; (ii) hydrogen sublattices in CaH_6_-, LaH_10_-, Ba_8_Si_46_- and Li_2_YH_17_-type superhydrides share the structure similarity to the known SOD-, AST-, MEP- and MTN-type zeolite frameworks, respectively.

**Figure 8.**
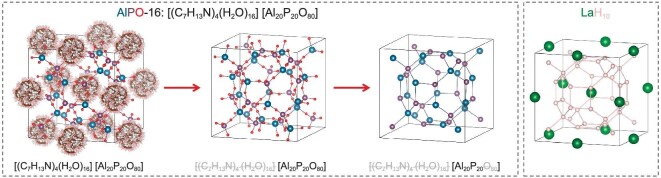
Comparison of the H sublattice in clathrate LaH_10_ and the AST-type zeolite framework in an AlPO_4_-16 material.

To understand how clathrate structures in superhydrides are related to zeolite frameworks, we take LaH_10_[17,18] as an example for the illustration (Fig. 8) whose hydrogen sublattice shares the structure similarity with the AST-type zeolite framework in an aluminum phosphate AlPO_4_-16 material [115]. It is noted that, when all non-framework cations are removed, AlPO_4_-16 turns out to be an open three-dimensional framework structure composed of corner-sharing TO_4_ (T = Al and P) tetrahedra (Fig. 8). The network of T atoms is identical to the hydrogen sublattice in LaH_10_.

The structure similarity between clathrate superhydrides and silicon-based clathrate or zeolite materials may give a hint on future design of high-*T*_c_ superconductors at ambient pressure. Encouragingly, there exists a large number of clathrate materials at ambient pressure that might be useful as templates for such a design. For example, over 200 zeolite framework types of material have been documented [116].

## CONCLUSION AND OUTLOOK

In this review, we provide an up-to-date perspective on the research field of superconducting clathrate superhydrides under high-pressure conditions. Clathrate superhydrides have emerged as the most promising candidates for hunting high-temperature superconductors. Finding room-temperature superconductors along this direction might become true in a near future.

Multi-element superhydrides are the immediate targets for the discovery of high-temperature superconductors. As the number of elements increase in hydrides, the number of conceivable structures and potential superconducting compounds grows rapidly [108,117], suggesting that there are more open rooms for clathrate superhydrides in ternary and quaternary systems than those in binary ones. However, the exponential growth of chemical space with increasing element species makes the search a great challenge both theoretically and experimentally. Therefore, it might be useful to summarize several strategies to accelerate the design of high-temperature superconducting superhydrides based on successful experiences: (i) doping/substituting metal elements into known clathrate superhydrides; (ii) introducing extra electrons via metal doping into known superhydrides that contain abundant quasi-H_2_ molecular units [110]; (iii) using small-radius elements to stabilize ternary hydrogen-based superconductors with alloy backbones [112]; (iv) atomic substitution of known clathrate materials at ambient pressure.

Given the rapid development of this field, we anticipate that certain aspects of this review might shortly become outdated. However, we trust that the methodological framework and the amalgamation of knowledge from both experiments and theories outlined here will provide a useful reference for future research and inspire exciting future discoveries.
